# Heart Rate Variability Can Detect Blunt Traumatic Brain Injury Within the First Hour

**DOI:** 10.7759/cureus.26783

**Published:** 2022-07-12

**Authors:** Min Zhu, Elizabeth E Blears, Claire B Cummins, Jordan Wolf, Omar A Nunez Lopez, Fredrick J Bohanon, George C Kramer, Ravi S Radhakrishnan

**Affiliations:** 1 Department of Surgery, University of Texas Medical Branch, Galveston, USA; 2 Surgery, Allegheny Health Network, Pittsburgh, USA; 3 Department of Anesthesiology, University of Texas Medical Branch, Galveston, USA; 4 Department of Pediatric Surgery, Children's Mercy Hospital, Kansas City, USA; 5 Department of Pediatric Surgery, Lane Regional Medical Center, Zachary, USA; 6 Department of Pediatric Surgery, University of Texas Medical Branch, Galveston, USA

**Keywords:** machine learning, hemorrhagic shock, trauma, traumatic brain injury, bovine model, heart rate variability

## Abstract

Introduction: In patients with multi-organ system trauma, the diagnosis of coinciding traumatic brain injury can be difficult due to injuries from the hemorrhagic shock that confound clinical and radiographic signs of traumatic brain injury. In this study, a novel technique using heart rate variability was developed in a porcine model to detect traumatic brain injury early in the setting of hemorrhagic shock without the need for radiographic imaging or clinical exam.

Methods: A porcine model of hemorrhagic shock was used with an arm of swine receiving hemorrhagic shock alone and hemorrhagic shock with traumatic brain injury. High-resolution heart rate frequencies were collected at different time intervals using waveforms based on voltage delivered from the heart rate monitor. Waveforms were analyzed to assess statistically significant differences between heart rate variability parameters in those with hemorrhagic shock and traumatic brain injury versus those with only hemorrhagic shock. Stochastic analysis was used to assess the validity of results and create a model by machine learning to better assess the presence of traumatic brain injury.

Results: Significant differences were found in several heart rate variability parameters between the two groups. Additionally, significant differences in heart rate variability parameters were found in swine within 1 hour of inducing hemorrhage in those with traumatic brain injury versus those without. These results were confirmed with stochastic analysis and machine learning was used to generate a model which determined the presence of traumatic brain injury in the setting of hemorrhage shock with 91.6% accuracy.

Conclusions: Heart rate variability represents a promising diagnostic tool to aid in the diagnosis of traumatic brain injury within 1 hour of injury.

## Introduction

Traumatic brain injury (TBI) is a serious public health problem with an estimated annual incidence of up to 500: 100,000 in the US and Europe [1]. TBI is a contributing factor in a third of all injury-related deaths with at least 1.7 million people seeking medical attention for TBI each year in the US [2]. Also, the incidence of TBI is rising in low- and middle-income countries, due to an increase in transport-related injuries [3]. In addition, the aging population in many countries has given rise to increasing numbers of elderly people who sustain TBI from falls [3].

Perhaps the most challenging scenario to diagnose TBI is in the setting of co-existing acute, severe hemorrhage [4]. With concomitant hemorrhage, TBI is difficult to diagnose because it is not always possible to perform imaging of the brain due to the need to address the primary source of hemorrhage within the thorax, long bones, or abdomen [5]. Moreover, signs of progression of TBI, such as irregular respirations or lack of motor tone, can also be present in hemorrhagic shock, confounding underlying TBI [4].

When patients are stabilized from hemorrhagic shock, patients often remain intubated and sedated, thereby precluding diagnosis by Glasgow Coma Scale (GCS) or neurological exam; therefore, imaging modalities, such as CT or MRI are typically used to assess the presence of TBI [4]. While CT and MRI are useful in identifying large intracranial hematomas, radiographic findings are not always present immediately after severe TBI [6]. Identifying severe TBI in the setting of hemorrhagic shock is essential because resuscitation, sedation, and goals of care are often managed differently depending on whether or not severe TBI is present [1-3]. In the absence of an early diagnostic tool that can work in parallel with the management of hemorrhagic shock, novel techniques for identifying TBI are being sought to improve the recognition of coinciding TBI to improve treatment protocols and outcomes in patients with multi-organ trauma that present with hemorrhagic shock.

Heart rate variability is determined by the sinoatrial node, which acts as a pacemaker for the myocardium [7]. The sinoatrial node is modulated by input from the parasympathetic and sympathetic branches of the autonomic nervous system, and these modulations result in variance in the R-R interval, also known as the “beat-beat interval” [7,8]. The R-R interval varies continuously, but this interval narrows with increased sympathetic tone leading to decreased heart rate variability under stressful conditions [9]. In addition, heart rate variability can be assessed rapidly using noninvasive electrodes [9]. Heart rate variability is a feasible correlate of increased intracranial pressure and other disruptions in the autonomic nervous system [7]. Heart rate variability has been previously shown to correlate with intracranial pressure, cerebral perfusion pressure, and clinical outcomes, though no studies have developed a model allowing for TBI diagnosis using heart rate variability [10]. A relationship between heart rate variability, hemorrhagic shock, and disruptions in the autonomic nervous system has also been established [11].

While previous authors have created separate swine models of heart rate variability to measure either hemorrhagic shock or severe TBI, this study is the first to examine heart rate variability in both conditions. With the heart rate variability parameters collected in this model, a machine learning technique called a Support Vector Machine was used to test whether patterns in heart rate variability could be used to differentiate between hemorrhagic shock injuries with and without TBI. We hypothesized that decreased heart rate variability could be associated with changes in hemodynamic patterns that are distinctive in the presence of TBI in hemorrhagic shock, and that the Support Vector Machine could provide an accurate model to describe decreased heart rate variability in the presence of TBI in acute hemorrhage in a preliminary porcine model.

## Materials and methods

Animals

Twelve female Yorkshire swine were used for this study based on the TBI model described by Manley et al., to induce severe TBI [12]. The swine had an average age of 6 months with an average weight of 40 kg. The swine were received in the animal facility and fed a standard diet for 14 days prior to experimentation to allow for acclimatization. Feeding was stopped 12 hours prior to the start of experimentation for the preparation of anesthesia. The project and all procedures were performed with the approval of the University of Texas Medical Branch Institutional Animal Care and Use Committee. This study was conducted in the large animal research facility at the University of Texas Medical Branch and animal care was provided according to the University of Texas Medical Branch’s Animal Resource Center and the National Research Council.

Procedure

A TBI model in swine was established which used a pneumatic bolt to deliver a severe, controlled TBI to generate tissue deformation and neuropathology similar to that seen in human TBI. Prior to the start of the procedure, all swine were randomized to receive either hemorrhage alone or hemorrhage with TBI. Animals were pre-medicated with 20-25 mg/kg ketamine, 1.2-2.8 mg/kg xylazine, 2.3-5.7 mg/kg zolazepam, and 0.3 mg buprenorphine for appropriate induction and maintained during the procedure with 1-2% isoflurane for appropriate sedation.

Heart rate monitoring electrodes were placed on the swine after induction of general anesthesia and placement in the supine position. A minute passed before the measurement of baseline heart rate variability, and swine were allowed to rest for 22 minutes to allow for heart rate stabilization. The TBI was then given to those swine who had been randomized to the TBI group. Blunt TBI was given using a pneumatic bolt and a pressure of 2.5-3.8 kg/cm^2^. The pneumatic bolt was positioned on the right side at the midpoint between the lambda suture posteriorly, the bregma suture anteriorly, the top of the cranium superiorly, and the base of the skull inferiorly [12]. Anesthesia was continued prior to delivery of pneumatic bolt trauma and a GCS of 3 was documented prior to delivery of the bolt trauma [12]. Administration of TBI took 10 minutes and the control group was rested during this time [12].

The femoral artery was used to hemorrhage the swine at 25 ml/kg for 5 minutes. With an average weight of 40 kg, the average hemorrhage per swine was approximately 1 l. Hemorrhage rates were verified using the Transonic Doppler flowmeter on the hemorrhage line. Hemorrhage was stopped, and heart rate variability continued to be monitored for an additional 5 minutes after hemorrhage. Anesthesia was maintained throughout the rest of the period, the TBI period, and the hemorrhage period with a confirmed GCS of 3. These phases were labeled as before TBI and hemorrhage (Tb), after TBI and during hemorrhage (Th), after TBI and hemorrhage (Tp), before hemorrhage alone (Hb), during hemorrhage (Hh), and after hemorrhage alone (Hp). After the hemorrhage recovery period of 5 minutes, anesthesia was weaned.

Signal analysis

The heart rate monitors produced signals within a spectrum of frequencies in Hertz (Hz). These frequencies were collected and analyzed by LabChart 8.0 (ADInstruments) to produce heart wave tracing based on the voltage delivered by the heart rate monitor. Using these tracings, signal peaks were produced during each of the three phases of the experiment.

**Figure 1:**
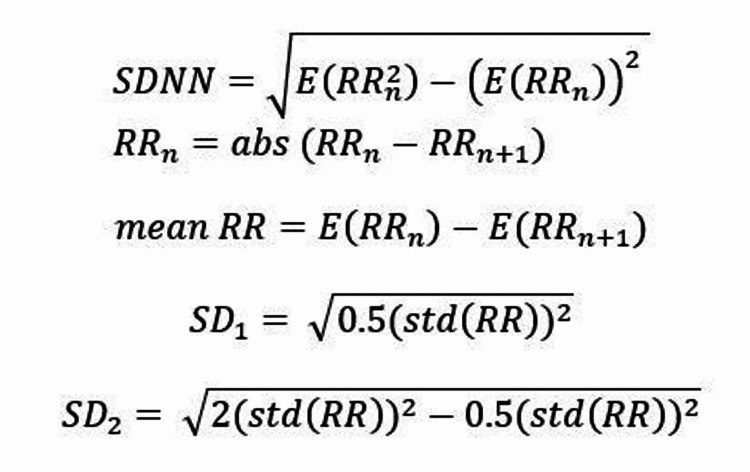
Formulas for RR, mean RR, SDNN, SD1, and SD2 in signal analysis. SDNN: standard deviation of the R-R intervals; SD1: non-linear dynamic values short-term variance SD2: long-term variance

R wave peaks were found using MATLAB 8.0 and Statistics Toolbox 8.1 (The MathWorks, Inc.) using filtering and half-wave cutting to label maximum and minimum R wave values and find subsequent R-R intervals (RR). Based on these peaks, time-domain statistical analysis was used to create matched curves. The mean heart rate (R), standard deviation of heart rate (SDR), RR, mean of the R-R intervals (mean RR), standard deviation of the R-R intervals (SDNN), and the non-linear dynamic values short-term variance (SD1), and long-term variance (SD2) were also calculated. The formulas for RR, mean RR, SDNN, SD1, and SD2 are shown in Figure 1.

RR interval refers to the time difference between each R wave peak, measured in milliseconds. The RR-RR interval refers to the differences between one RR interval and the next, also measured in milliseconds. The RR-RR interval is presented as between 1 and 10 milliseconds, 10 and 20 milliseconds, 20 and 30 milliseconds, 30 and 40 milliseconds, 40 and 50 milliseconds, or greater than 50 milliseconds. The percentage of RR intervals (pNN) was then expressed as “pNNc” where “c” represents the category examined.

Statistical analysis

The eight parameters R, SDR, RR, mean RR, SDNN, SD1, SD2, the ratio SD1/SD2, and pNN for all time categories (pNN1-10, pNN10-20, pNN20-30, pNN30-40, pNN40-50, and pNN50) were calculated for each of the six time points (Tb, Th, Tp, Hb, Hh, and Hp) using MATLAB. Data sets were checked for normality using histogram plots. Statistical significance was set for all tests with α= 0.05, and groups were analyzed using either Student’s t-tests or one-way analysis of variance, depending upon the size of the groups. RR values in the highest 2.5% and lowest 2.5% of all values collected were removed to reduce interference due to outliers generated by the heart monitor. All error bars in the figures were representative of the standard error of the mean. Statistical significance was denoted as follows: “*” for P<0.05. To verify these results, stochastic analysis was performed. For each of the 12 instances of statistical significance identified above, swine were randomly assigned to two groups of six swine in each. Repeated t-tests were performed 100 times and the number of statistically significant instances was noted, producing a distribution test. This distribution test was then repeated 10 times.

Poincaré plots, also known as the scatter plots, first return maps, and Lorenz plots, are both a useful visual tool to summarize an entire RR time series and a quantitative technique that gives non-linear information on the long- and short-term heart rate variability [13,14]. Poincaré plots were performed for each of the six groups (Hb, Hh, Hp, Tb, Th, and Tp) to detect the presence of oscillation in non-linear dynamic systems. These plots were generated by plotting RRn on the x-axis and RRn+1 on the y-axis.

Model generation

Statistically significant parameters were confirmed via stochastic analysis and then used to generate a prediction model. Non-significant parameters were labeled accordingly and excluded. Of those that were statistically significant, all possible combinations of parameters were generated to find the most accurate model and then tested for accuracy. To test for accuracy, the appropriate parameters for each swine were chosen and sorted into control and TBI groups. One measurement was randomly chosen as the test data for each parameter in the model, while the remaining measurements were used as training data. Training data was used with the Library Support Vector Machine (LIBSVM) tool to generate a linear model and save each parameter in every model [15]. A mean model was then generated by using the information generated during these recurring tests. The mean model was subsequently tested by using the previously designated test data and repeated 100 times to obtain an average accuracy.

## Results

Heart rate variability parameter differences between traumatic brain injury and no injury groups

**Figure 2:**
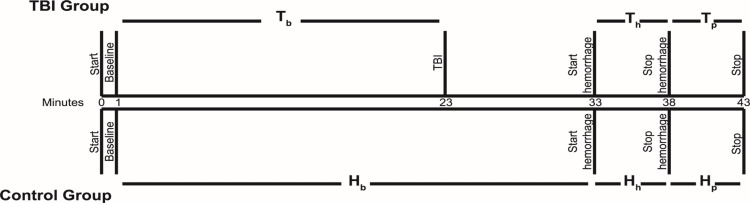
Timeline of TBI and hemorrhage model protocol with experimental groups labeled. TBI: traumatic brain injury

**Figure 3:**
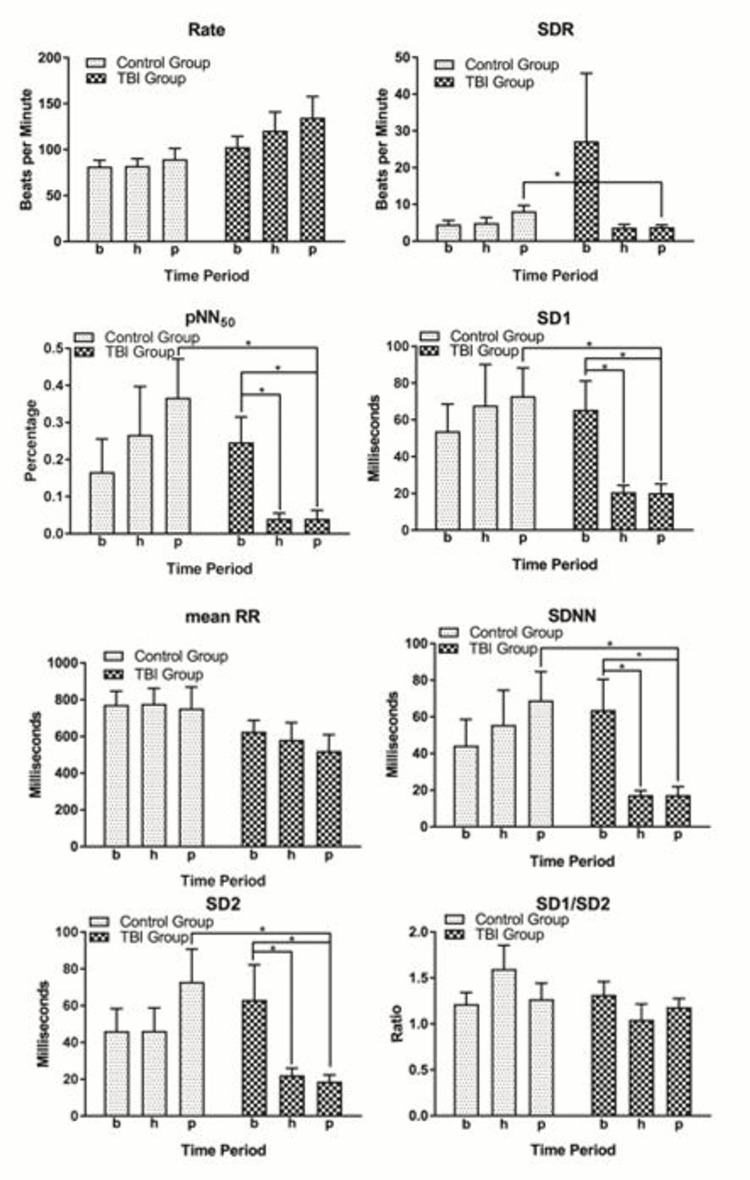
Heart rate variability parameter differences. Bars represent the mean of each group with the error bars representing standard error of the mean. Parameters examined include heart rate, standard deviation of heart rate (stR), RR interval (RR), standard deviation of RR interval (SDNN), percentage of RR intervals greater than 50 ms (pNN50), and the non-linear dynamic values short-term variance (SD1), long-term variance (SD2), and the ratio SD1/SD2. Significance is denoted as *P<0.05.

A timeline of the procedure is presented in Figure 2. Since the TBI was induced prior to the hemorrhage, there was a control period where TBI alone was compared to no injury (Figure 2, Minute 23-33), which did not demonstrate differences in heart rate variability parameters between the two groups (Figure 3). Therefore, the changes in heart rate variability with hemorrhage were noted to be due to the combination of these two physiologically challenging conditions, rather than TBI alone. Of the eight parameters examined, five parameters demonstrated statistically significant changes: SDR, SDNN, pNN50, SD1, and SD2 (Figure 3). Between the hemorrhage alone and hemorrhage with TBI groups, SDR (Hp vs Tp, P<0.05), SDNN (Hp vs Tp, P<0.05), pNN50 (Hp vs Tp, P<0.05), SD1 (Hp vs Tp, P<0.05), and SD2(Hp vs Tp, P<0.05) were all significantly different.

Heart rate variability parameter differences within traumatic brain injury group

Statistically significant differences in heart rate variability were also present over the time course of the experiment, demonstrating decreased heart rate variability during the TBI (as shown in Figure 3). Within the TBI group, SDNN (Tb vs Th, P<0.05 and Tb vs Tp, P<0.05), pNN50 (Tb vs Th, P<0.05 and Tb vs Tp, P<0.05), SD1 (Tb vs Th, P<0.05 and Tb vs Tp, P<0.05), and SD2 (Tb vs Th, P<0.05 and Tb vs Tp, P<0.05) were all significantly different. Notably, these changes were seen as early as 15 minutes after TBI in several parameters. No significant differences were noted between the baseline parameters of either group (Hb vs Tb) or within the control group (Hb vs Hh and Hb vs Hp). All differences were noted either between the control group and the TBI group or within the TBI group (Hp vs Tp, Tb vs Th, and Tb vs Tp). SDNN, pNN50, SD1, and SD2 are features of the RR-RR interval, and SDR is a feature of the RR interval. This indicates that the most important heart rate variability parameter following TBI is the RR-RR interval, with the RR interval as a secondary parameter.

Stochastic analysis

After stochastic analysis, differences occurred at a probability of 1.4%, which was below our defined level of statistical significance (5%). Therefore, our results represent true statistical significance, even within this small sample size.

Poincaré plot and RR-RR interval percentage distribution

**Figure 4:**
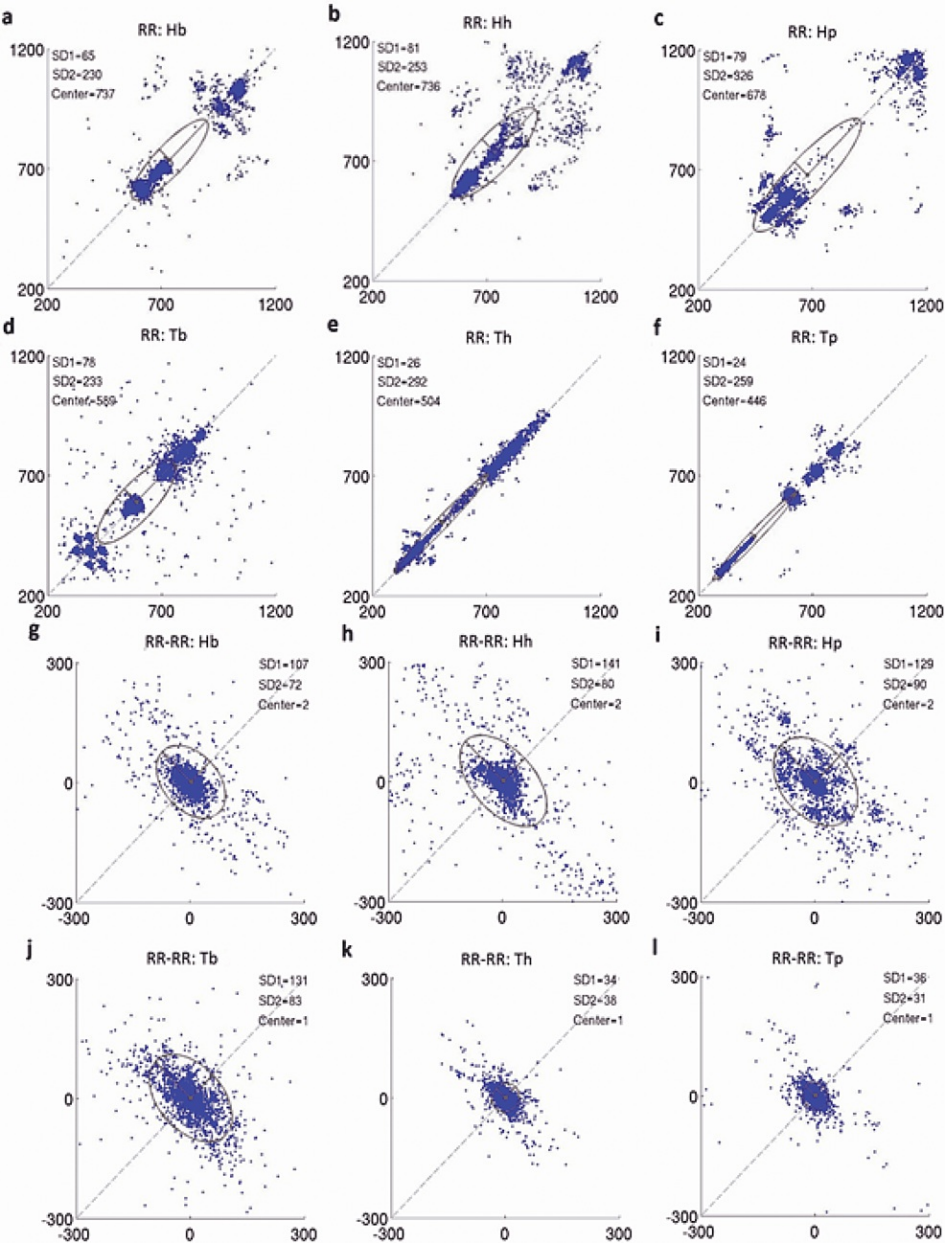
Poincaré Plot analysis. The first 6 plots represent plotting RRn on the x-axis and RRn+1 on the y-axis. The second 6 plots represent plotting the difference between RRn and RRn+1 on the x-axis and RRn+1 RRn+2 on the y-axis.

Poincaré plots were performed for each of the six groups (Hb, Hh, Hp, Tb, Th, and Tp) as shown in Figure 4. Loss of heart rate variability was detected after TBI as seen by the very narrow clustering of points in the Th and Tb groups. No remarkable changes were seen between the control groups (Hb, Hh, and Hp) or between the baseline groups (Hb and Tb), which was consistent with our previous results.

**Figure 5:**
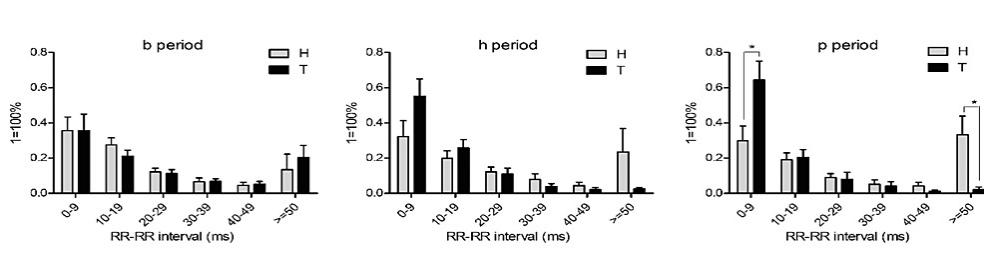
RR-RR interval percentage distribution. Each bar represents the percentage of RR-RR intervals which fell into each 10 millisecond time range. Bars represent the mean of each group with the error bars representing standard error of the mean. Significance is denoted as *P<0.05.

The absolute value of the difference between the RR intervals, as shown in Figure 5, demonstrated the RR-RR interval percentage distribution. No significant differences were seen between the control and TBI groups in the baseline period in the percentage of RR-RR intervals in each 10-millisecond interval bin. A bimodal distribution was present, with the majority of RR-RR intervals taking place between 0 and 9 milliseconds, 10 and 19 milliseconds, and ≥ 50 milliseconds. In the post-hemorrhagic and TBI groups, a statistically significant difference was seen between the control and TBI groups. Swine with TBI had significantly more RR-RR intervals between 0 and 9 milliseconds and significantly fewer RR-RR intervals ≥ 50 milliseconds. The TBI group no longer followed a bimodal distribution, and the majority of RR-RR intervals in the Tp group were between 0 and 9 milliseconds.

Model results

**Figure 6:**
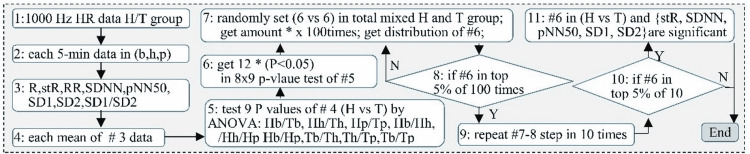
Method used to find significant parameters (*), and build the prediction model (SDR, SDRNN, pNN50, SD1, and SD2 were found to be significant after stochastic analysis). HR: heart rate; SDNN: standard deviation of RR interval; pNN50: percentage of RR intervals greater than 50 ms; the SD1: non-linear dynamic values short-term variance; SD2: long-term variance; SDR: standard deviation of heart rate

Stochastic analysis revealed confirmed statistically significant differences in SDR, SDNN, pNN50, SD1, and SD2 as shown in Figure 6.

**Figure 7:**
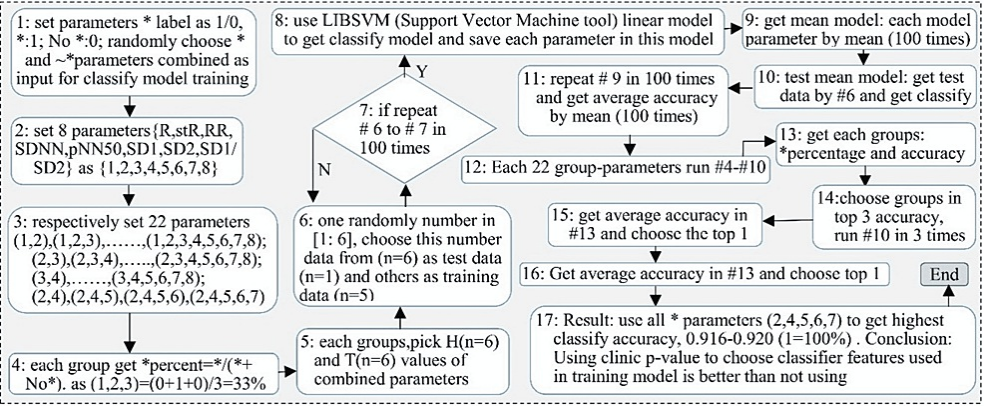
Predictive model created using LIBSVM software, training data, and each of these parameters was numbered as follows: 1-R, 2-SDR, 3- mean RR, 4-SDNN, 5-pNN50, 6-SD1, 7-SD2, 8-ratio SD1/SD2. SDNN: standard deviation of RR interval; pNN50: percentage of RR intervals greater than 50 ms; the SD1: non-linear dynamic values short-term variance; SD2: long-term variance; SDR: standard deviation of heart rate

These parameters were used to generate a prediction model using the significant parameters with the methodology depicted in Figure 7. The remaining parameters, R, mean RR, and the ratio SD1/SD2 were taken as non-significant in the model generation. Twenty-two possible combinations of parameters were generated, and each possible model was tested for accuracy, with one measurement randomly chosen as the test data, and the other five measurements used as training data.

Results of the accuracy testing are depicted in Table 1. Overall, as the percentage of significant parameters increased, the model accuracy increased. The greatest accuracy was seen with the model which employed only the five parameters that were designated to be significant (SDR, SDNN, pNN50, SD1, and SD2). The model utilizing these five parameters was found to be 91.8% accurate at determining whether a swine had received a TBI in addition to hemorrhage.

**Table 1:** Average accuracy of models with combined parameters at predicting TBI. Parameters are numbered as follows: 1-R, 2-SDR, 3- mean RR, 4-SDNN, 5-pNN50, 6-SD1, 7-SD2, 8-ratio SD1/SD2.  The percentage of parameters that demonstrated significant changes is given by columns with the average accuracy of those models. SDR: standard deviation of heart rate; SDNN: standard deviation of RR interval; pNN50: percentage of RR intervals greater than 50 ms; the SD1: non-linear dynamic values short-term variance; SD2: long-term variance; SDR: standard deviation of heart rate

Parameters	Accuracy (%)	Parameters	Accuracy (%)	Parameters	Accuracy (%)	Parameters	Accuracy (%)
1,2	84.9	2,3	84.8	3,4	63.9	2,4	91.3
1-3	76.4	2-4	82.1	3-5	75.2	2,4,5	91.4
1-4	78.9	2-5	84.9	3-6	74.3	2,4,5,6	91.8
1-5	79.0	2-6	84.5	3-7	76.6	2,4,5,6,7	91.6
1-6	79.4	2-7	82.3	3-8	75.0		
1-7	78.8	2-8	79.1				
1-8	76.5						
Percent Significant (%)	Average Accuracy (%)	Percent Significant (%)	Average Accuracy (%)	Percent Significant (%)	Average Accuracy (%)	Percent Significant (%)	Average Accuracy (%)
50.8	79.1	68.3	82.9	78.7	73.1	100	91.5

## Discussion

While heart rate variability has been explored in the setting of TBI, this study is the first to demonstrate its utility in a swine model with an accompanying injury. Early diagnosis of TBI represents an important area for potential improvement in trauma-related morbidity and mortality. TBI is difficult to diagnose quickly and imaging techniques cannot always confirm its presence or its progression [5,16]. Heart rate variability represents a noninvasive tool that could be used to identify patients with TBI quickly after injury, thereby opening the possibility of early treatment and better outcomes. Our results demonstrated that statistically significant changes were seen in SDR, SDNN, pNN50, SD1, and SD2 after TBI in the setting of hemorrhage. Moreover, these results were tested with stochastic analysis to validate accuracy.

Additionally, Poincaré plot analysis demonstrated a loss of heart rate variability after TBI and significantly more RR-RR intervals between 0 and 9 milliseconds, which corroborates the utility of this parameter in distinguishing the presence of TBI during hemorrhagic shock. Additionally, this study is the first to generate a model which associates TBI in the setting of hemorrhage with 91.8% accuracy.

Since the initial reports of recording heart rate variability parameters from Wolf et al. in 1978, there has been significant progress in the knowledge surrounding heart rate variability [17]. Heart rate variability parameters can be split into time-domain parameters and frequency-domain parameters. Time-domain parameters include those studied in this paper, such as SDNN and pNN50, while frequency-domain parameters include very low frequency, low frequency, and high frequency [18]. A reduction in time-domain parameters, particularly SDNN, is considered indicative of diminished vagal and increased sympathetic modulation of the sinoatrial node, while frequency-domain parameters describe the periodic oscillation patterns over an extended period, such as 24 hours [19]. For 10 minutes prior to inducing hemorrhage, there were no changes in either frequency or time-domain parameters, which was different from prior studies in humans and swine [20,21]. Therefore, the changes in heart rate variability with hemorrhage were noted to be due to the combination of these two physiologically challenging conditions, rather than TBI alone, and could increase the value of the model in associating TBI with coinciding hemorrhage.

This study can add to the established literature on heart rate variability since this study demonstrated a significant reduction in SDR, SDNN, and pNN50 after TBI when compared to the hemorrhage control group. In addition, there was a significant decrease over time in SDNN and pNN50 from their baseline values after TBI. This could correlate with the suspicion that others have had that a significant progressive decrease in vagal activity occurs after TBI [22]. Findings in animals and humans demonstrate that cardiac parasympathetic blockade eliminates all high-frequency heart fluctuations and 75% of low frequency and very low-frequency fluctuations while sympathetic blockade has no effect on high-frequency fluctuations [23]. Therefore, the results of this study were more likely the result of the vagal blockade that is suspected of occurring alongside TBI, in keeping with previous studies. Interestingly, no significant changes were observed in any parameters after hemorrhage alone, indicating that heart rate variability may prove to be an important tool for recognizing TBI in those without hemorrhagic shock.

Poincaré plots were also significant for parameters that could be used to distinguish between the two groups. Quantitative analysis of the plots has been reported as a surrogate for time-domain analysis of heart rate variability, and analysis of the geometric patterns has been used to generate programs that can automatically detect cardiac arrhythmias [24]. Two descriptors, SD1 and SD2, are typically used to describe Poincaré plots; SD1 is the dispersion of the plot across the line of identity (y=x) and describes instant beat-to-beat variability of RR intervals while SD2 is the dispersion along this line and is related to long-term heart rate variability [13]. Our results demonstrate a significant decrease in both SD1 and SD2 between the hemorrhage and the TBI groups. Additionally, there was a significant decrease in SD1 and SD2 over time in the TBI group. However, the SD1/SD2 ratio was preserved without change in all groups. This indicated that there was a concomitant decrease in both short- and long-term variability after TBI.

Visually, the Poincaré plots demonstrated much tighter grouping after TBI, which was representative of these results. Absolute RR interval differences were closely related to Poincaré plots and also demonstrated statistically significant changes. After TBI, the absolute RR interval differences no longer followed a bimodal distribution, and the majority of RR-RR intervals after TBI were between 0-9 milliseconds. In theory, real-time generation of heart rate variability parameters and Poincaré plots on a bedside monitor could allow practitioners to potentially assess hemodynamic parameters that are associated with the presence of co-existing TBI in hemorrhagic shock.

Due to advances in sensor technology and computing power, real-time capture and assessment of high-frequency data is now a viable option for trauma patients. In order to use this high-fidelity data to achieve more accurate diagnostic capabilities, the development of machine learning technologies is being utilized to efficiently process large and/or heterogeneous data sources. Several studies have demonstrated the feasibility of applying machine learning techniques to the assessment of heart rate variability in a variety of conditions [25-27]. Our model was able to successfully determine the likelihood of TBI in the setting of hemorrhage using heart rate variability with 91.8% accuracy within 1 hour of the injury. The accuracy of the model improved as the percentage of significant factors used in its generation increased, which is consistent with other machine learning models [28,29]. The optimal model used only four out of the five significant parameters; however, using all five significant parameters still generated a model with 91.6% accuracy.

Despite the accuracy of this data, it is not clear how generalizable swine models of TBI are to humans. They are particularly apt at simulating the sequence of events in human TBI due to the similarities in brain composition, such as the distribution of gyri and white and grey matter, and swine models have also been used in studies characterizing the heart rate variability response to hemorrhagic shock [21,30]. Despite this, human trauma populations represent a very heterogeneous group of ages, causes, courses, and consequences, which are difficult to model and represent a limitation to the study. Additionally, this model only accounts for one type of TBI in one cranial location. And it is possible that TBI in other cranial locations may have different findings, meriting further study.

## Conclusions

The identification of TBI in patients with co-existing hemorrhagic shock after blunt trauma represents one of the most challenging diagnoses, due to the inability to obtain rapid cranial imaging while often treating coinciding life-threatening injuries, and lack of overt symptoms on physical exam as well as the non-specificity of vital signs and laboratory work. Heart rate variability represents a promising new diagnostic tool for the early diagnosis of TBI in the setting of hemorrhagic shock. Poincaré plots and a stochastic analysis were able to generate a robust model from a limited swine model, the closest available to humans, with approximately 91.8% accuracy. It is possible that the usage of heart rate variability with machine learning tools could be used to provide more accurate diagnosis of this common combination of injuries.
